# Establishment and Validation of a Nomogram Prognostic Model for Epithelioid Hemangioendothelioma

**DOI:** 10.1155/2022/6254563

**Published:** 2022-10-06

**Authors:** Yujun Li, Zibo Zhang, Chunxia Zhang, Jia Li, Bin Zhang, Yan Dong, Xiaonan Cui

**Affiliations:** ^1^The First Affiliated Hospital of Dalian Medical University, Dalian, China; ^2^Department of Oncology, The First Affiliated Hospital of Dalian Medical University, Dalian, China

## Abstract

**Background:**

Epithelioid hemangioendothelioma (EHE) is an ultrarare vascular sarcoma. At present, the epidemiological and clinical characteristics and prognostic factors are still unclear. Our study attempted to describe clinical features, investigate the prognostic indicators, and establish the nomogram prediction model based on the Surveillance, Epidemiology, and End Results (SEER) database for EHE patients.

**Methods:**

The patients diagnosed with EHE from 1986 to 2018 were collected from the SEER database and were randomly divided into a training group and a validation group at a ratio of 7 : 3. The Cox proportional hazard models were used to determine the independent factors affecting prognosis and establish a nomogram prognostic model to predict the survival rates for patients with EHE. The accuracy and discriminative ability of the model were measured using the concordance index, receiver operating characteristic curves, and calibration curves. The clinical applicability and application value of the model were evaluated by decision curve analysis.

**Results:**

The overall age-adjusted incidence of EHE was 0.31 patients per 1,000,000 individuals, with a statistically significant difference per year. Overall survival at 1, 5, and 10 years for all patients was 76.5%, 57.4%, and 48.2%, respectively. Multivariate Cox regression analysis identified age, tumour stage, degree of tissue differentiation, surgical treatment, chemotherapy, and radiotherapy as independent factors affecting prognosis (*P* < 0.05). The C-index values for our nomogram model of training group and validation group were 0.752 and 0.753, respectively. The calibration curve was in good agreement with the actual observation results, suggesting that the prediction model has good accuracy. The decision curve analysis indicated a relatively large net benefit.

**Conclusions:**

The nomogram model may play an important role in predicting the survival rate for EHE patients, with good concordance and accuracy, and can be applied in clinical practice.

## 1. Introduction

Epithelioid hemangioendothelioma (EHE) is an ultrarare vascular sarcoma, usually behaving as a low-grade malignancy despite a high propensity for systemic involvement [1]. EHE was first named by Professors Weiss and Enzinger in 1982 and was included as a malignant tumour in the 4th edition of the World Health Organization (WHO) classification of soft tissue tumours in 2013 [2, 3]. The degree of uncertainty in selecting the most appropriate treatment of EHE patients is high, treatment options vary, and the adoption of new treatments is inconsistent across the world, resulting in suboptimal outcomes for many patients. EHE does not have specific symptoms and signs in clinical practice. The initial diagnosis can be incidental in completely asymptomatic patients. The symptoms are pain (40%), a palpable mass (6%-24%), and weight loss (9%). Although reports on EHE have increased in recent years, they are limited to case reports and retrospective studies with small sample sizes. The epidemiological and clinical characteristics and prognostic factors for EHE are still unclear.

The National Cancer Institute (NCI)–Surveillance, Epidemiology, and End Results (SEER) database (referred to as the SEER database) is a commonly used public database that contains information on the incidence, mortality, and prevalence of cancers in some states and counties in the United States (approximately 35% of the U.S. population). The data are from large medical institutions, individual private clinics, laboratories, autopsy results, etc., and are regularly updated with retrospective clinical data, patient demographics, primary tumour site, tumour morphology, diagnosis stage, treatment course, and survival status [4]. Due to its large data sample size, wide range of resources, and good statistical results, studies based on the SEER database have good clinical reference significance, especially with regard to rare diseases.

Traditionally, TNM staging or MSTS/Enneking surgical staging has been used to evaluate the prognosis of soft tissue tumours. Unfortunately, the prognosis of different types of soft tissue tumours is often different even at the same stage. Especially as EHE, the existing staging methods are not enough to make treatment choices and prognosis judgments. A nomogram is a prediction tool that can be used to visualize the results of a multivariate Cox regression model, using multiple clinical indicators and line segments labeled with different scores to create statistical predictions, and is a simple graphical display of models that calculate the probability of occurrence of a certain clinical event or outcome [5]. Currently, nomograms are widely used to predict the prognosis of cancer patients. For many cancers, nomograms have been proven to be superior to the traditional TNM staging system, serving a valid alternatives and even being proposed as new standards [6].

Our study attempts to establish the nomogram prediction model based on the SEER database for EHE patients. The model involves independent prognostic factors that affect EHE patient survival, in order to provide clinicians with survival and prognosis help for personalized treatment of patients with EHE.

## 2. Materials and Methods

### 2.1. Data Sources

The SEER∗Stat software (version number: 8.3.9) was downloaded from the official website of the SEER database (https://seer. cancer. gov) to extract the screened case-related data from the database updated in March 2021. The earliest diagnosis of EHE registered in the SEER database was in 1986, Therefore, a total of 512 patients with histologically confirmed EHE from 1986 to 2018 were screened under “Case Listing Session” mode in the SEER∗Stat software for prognostic factor analysis. The inclusion criteria were as follows: time of diagnosis from 1986 to 2018 and the International Classification of Diseases for Oncology (3rd edition, ICD-O; issued by the WHO) code 9133 (EHE), with the biological behaviour listed as “malignant.” By analyzing the clinical characteristics of the included cases, we found that the incidence of EHE has increased significantly since 2000, so a total of 682 patients from 2000 to 2018 were selected under “Rate Session” model for incidence correlation analysis.

After meeting the above inclusion criteria, the following exclusion criteria were applied: no clear survival time and multiple primary tumour locations. Because the SEER only contains deidentified data, approval by an institutional review board was not required for this study.

### 2.2. Extraction of Clinical Characteristic Variables

Using the SEER database, the data for the following clinical characteristic variables were collected: sex, age at diagnosis, year of diagnosis, race, primary tumour location, tumour histological grade, tumour stage, treatment method (surgery, chemotherapy, and radiotherapy), survival time or last follow-up time, survival status, and cause of death. The patients were divided by sex into 2 groups: “male” and “female.” Age at diagnosis was a continuous variable. The patients were divided by race into 3 groups: “black,” “white,” and “other (Asian).” The patients were divided into 5 groups based on primary tumour location: “head and neck,” “lung,” “liver,” “bone and soft tissue,” and “other.” The patients were divided by tumour histological grade into 3 groups: “good differentiation” (grades I and II), “poor differentiation” (grades III and IV), and the “unknown differentiation.” Patients were divided by tumour stage into 4 groups: “localized,” “regional,” “distant,” and “unknown.” “Localized” was defined as a focal lesion characterized by a single tumour lesion, “regional” was defined as a single organ with multifocal involvement or a single lesion with regional lymph node involvement, and “distant” was defined as multiorgan with distant metastatic involvement. The patients were divided by surgical treatment received into 3 groups: “surgical treatment,” “no-surgical treatment,” and “unknown.” The patients were divided by chemotherapy received into 2 groups: “chemotherapy” and “no-chemotherapy.” Last, the patients were divided by radiotherapy received into 2 groups: “radiotherapy” and “no-radiotherapy”.

### 2.3. Primary Study Endpoints

The main study endpoints were overall survival (OS) and cancer-specific survival (CSS). The last follow-up time was defined as March 2021, the date of the last update of the SEER database prior to this study. OS was defined as the time from the date of diagnosis to the death of the patient due to any reason or the last follow-up. CSS was defined as the time from the date of diagnosis to the death of the patient due to the tumour. Patients who were still alive at the time of the last follow-up were defined as censored survival data.

### 2.4. Statistical Analysis

**Figure 1:**
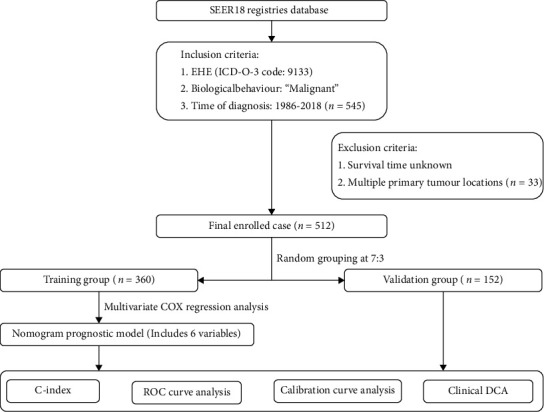
Overall workflow describing the process used to establish and validate the prognostic evaluation model of predict prognostic outcomes (OS).

The SPSS 25.0 software was used for the statistical analysis. The Kaplan-Meier method was used for the survival analysis, and incidence trends and survival curves were plotted. The log-rank test was performed for the univariate analysis; variables that were statistically significant in the univariate analysis were included in a Cox proportional hazard regression model for a multivariate analysis to determine independent factors affecting OS. *P* < 0.05 was considered statistically significant. R version 4.1.2 and the extension packages RMS, Foreign, Survival, Caret, ggDCA, and timeROC were used to generate the prediction model. The concordance index (C-index), calibration curves, receiver operating characteristic (ROC) curves, and prognostic decision curve analysis (DCA) were used to evaluate the accuracy and discriminative ability of the prediction model. The C-index is similar to the area under the ROC curve (AUC); the higher is the value, the higher is the prediction accuracy, and the closer is the prediction risk to the standard curve, the higher is the fitness of the model. The overall workflow describing the process used to establish and validate the prognostic evaluation model of predict prognostic outcomes (OS) is shown in Figure 1.

## 3. Results

### 3.1. Age-Adjusted Incidence of EHE

**Figure 2:**
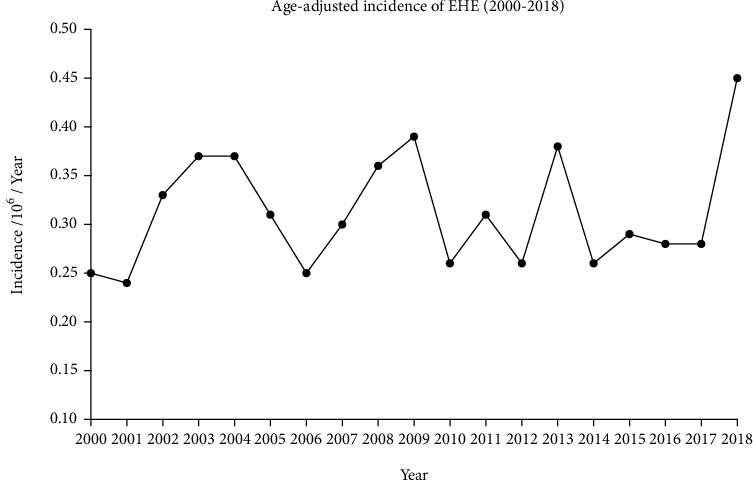
Age-adjusted incidence of EHE.

From the SEER-21 database, 682 patients were selected from 2000 to 2018. The incidence of EHE was 0.31 patients per 1,000,000 individuals, with a statistically significant difference per year. The incidence was generally higher among females than males. The APC was 0.52% (95% CI, -1.16%-2.24%; *P* < 0.05) (Figure 2).

### 3.2. Patient Characteristics

**Figure 3:**
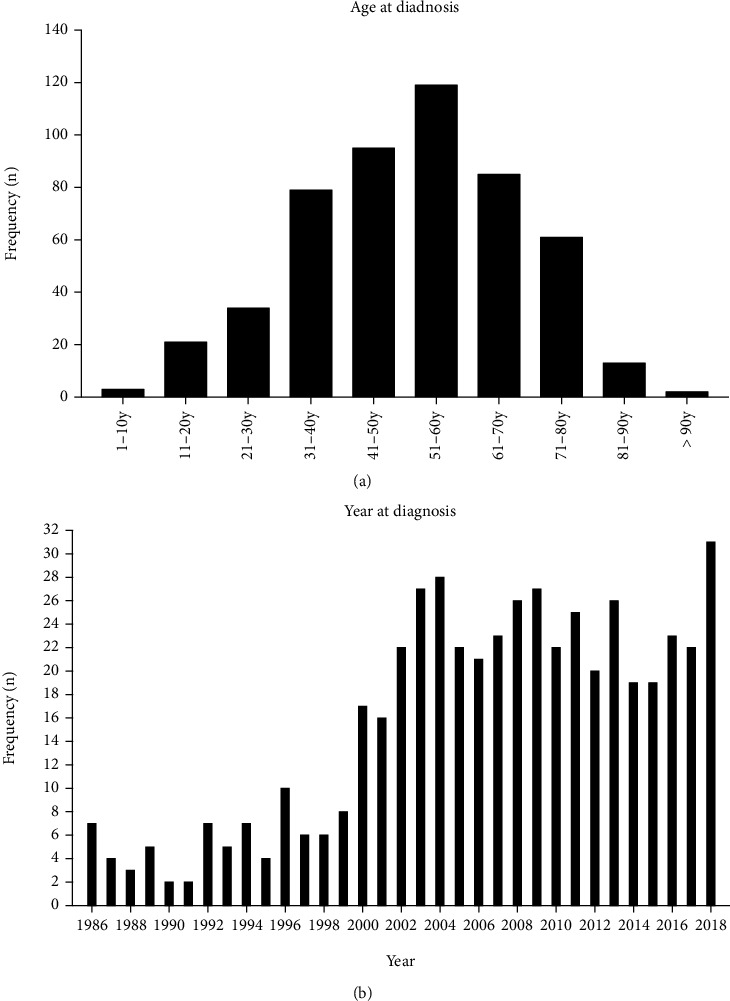
(a) Age at diagnosis (EHE patients). (b) Year of diagnosis (EHE patients).

Using the established inclusion and exclusion criteria, this study screened a total of 512 EHE cases from the relevant databases from 1986 to 2018. The average age of the patients was 50.79 ± 17.076 years (range 2-92 years). Compared with that in the 1990s, the number of confirmed patients increased significantly after 2000 (Figure 3(a)). There were more female patients (300; 58.9%) than male patients. Patients aged 50-59 years accounted for the largest proportion (119; 23.2%) (Figure 3(b)). There were 420 white patients (82.0%). The most common primary tumour location was the liver (131; 25.6%), followed by the lung and bone (soft tissue) (124; 24.2% and 121; 23.6%, respectively). Most patients were diagnosed with distant organ metastasis (187; 36.5%). The histological grade of most patients (378; 73.8%) was not clear. Among the definite tumour grades, 87 cases (17.0%) show a good differentiation. More than half of the patients received surgical treatment (263; 51.4%), and a small number of patients received chemotherapy and radiotherapy (139; 27.1% and 120; 23.4%, respectively). There were no statistically significant differences in age, sex, race, degree of tissue differentiation, tumour stage, primary tumour location, use/no-use of surgery, use/no-use of chemotherapy, use/no-use of radiotherapy, and survival between the training group and the validation group (*P* > 0.05). The sample distribution was uniform. The general clinical characteristics of the enrolled patients are provided in Table 1.

### 3.3. Survival Prognosis Analysis of Patients with EHE

**Figure 4:**
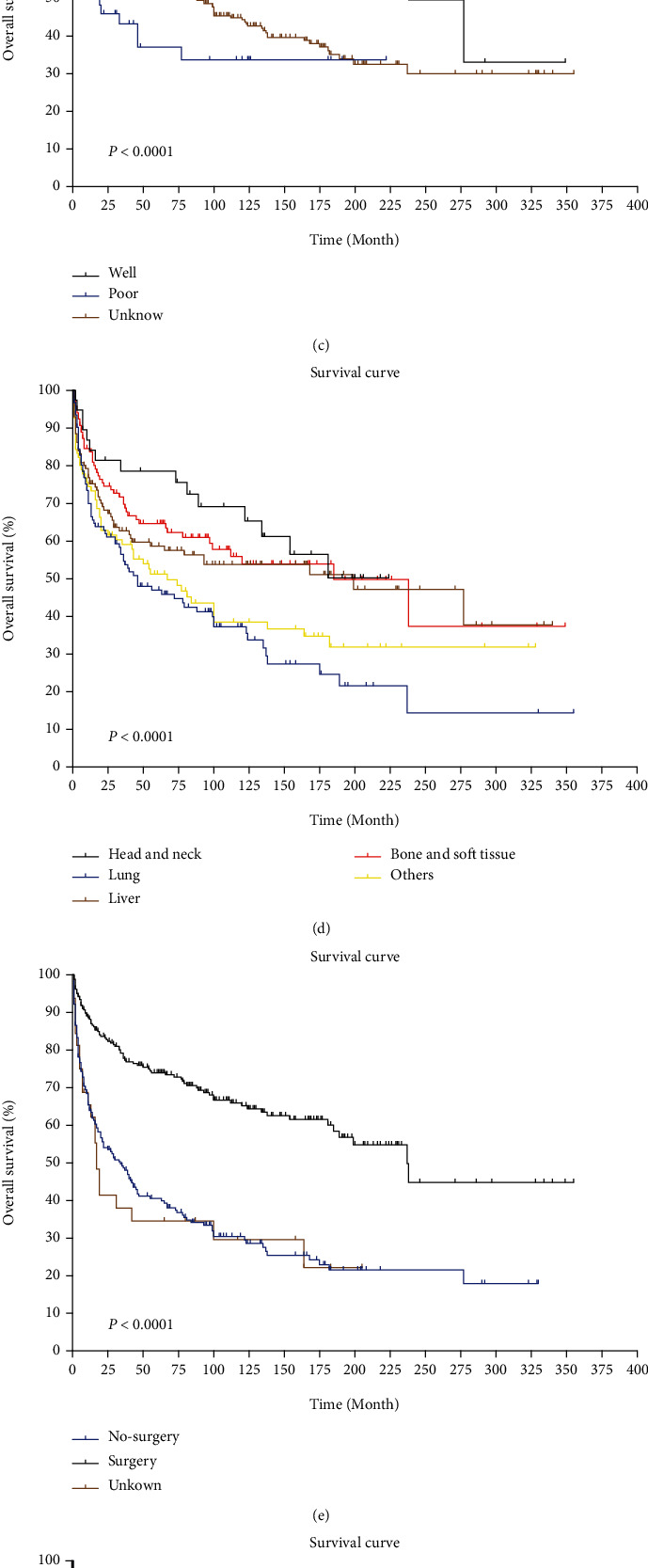
(a) Overall survival of all patients. (b) Survival analysis for staging. (c) Survival analysis for grade. (d) Survival analysis for primary tumour location. (e) Survival analysis for surgery. (f) Survival analysis for chemotherapy. (g) Survival analysis for radiotherapy.

The median survival time for all patients with EHE was 8.3 years (100 months), and the longest survival time was greater than 29.5 years. The 1-year, 5-year, and 10-year OS rates were 76.5%, 57.4%, and 48.2%, respectively, and the corresponding CSS rates were 77.3%, 59.0%, and 52.4%, respectively. The data of CSS was similar to those of OS. The survival rates for patients are detailed in Table 2. Kaplan-Meier and log-rank test results suggested that clinical stage, degree of differentiation, primary tumour location, and treatment method (surgery, chemotherapy, and radiotherapy) were associated with prognosis (*P* < 0.05). The survival curses are shown in Figures 4(a)–4(g).

### 3.4. Univariate and Multivariate Analyses of Patients in the Training Set

According to the results of the univariate and multivariate Cox proportional hazard regression models, age, poor tissue differentiation, late clinical staging, chemotherapy, and radiotherapy were independent factors for a poor prognosis, and surgical treatment was an independent factor for a good prognosis (*P* < 0.05) (Table 3).

### 3.5. Construction of a Clinical Prognostic Model of EHE

**Figure 5:**
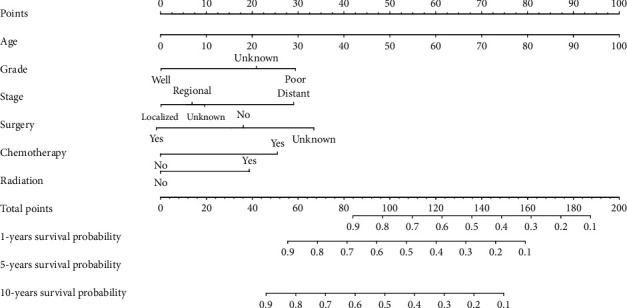
Prediction of 1-, 5-, and 10-year OS rates for EHE patients using the nomogram model.

The 6 independent prognostic factors (age, degree of tissue differentiation, stage, surgical treatment, chemotherapy, and radiotherapy) obtained in the multivariate Cox regression analysis were selected to establish a nomogram prognostic model, and each variable had its own dependent variables. The scores for each variable were added and used as the total score to ultimately predict the 1-year, 5-year, and 10-year survival rates for patients with EHE (Figure 5).

### 3.6. Validation of the Clinical Prognostic Model of EHE and Its Clinical Applicability

**Figure 6:**
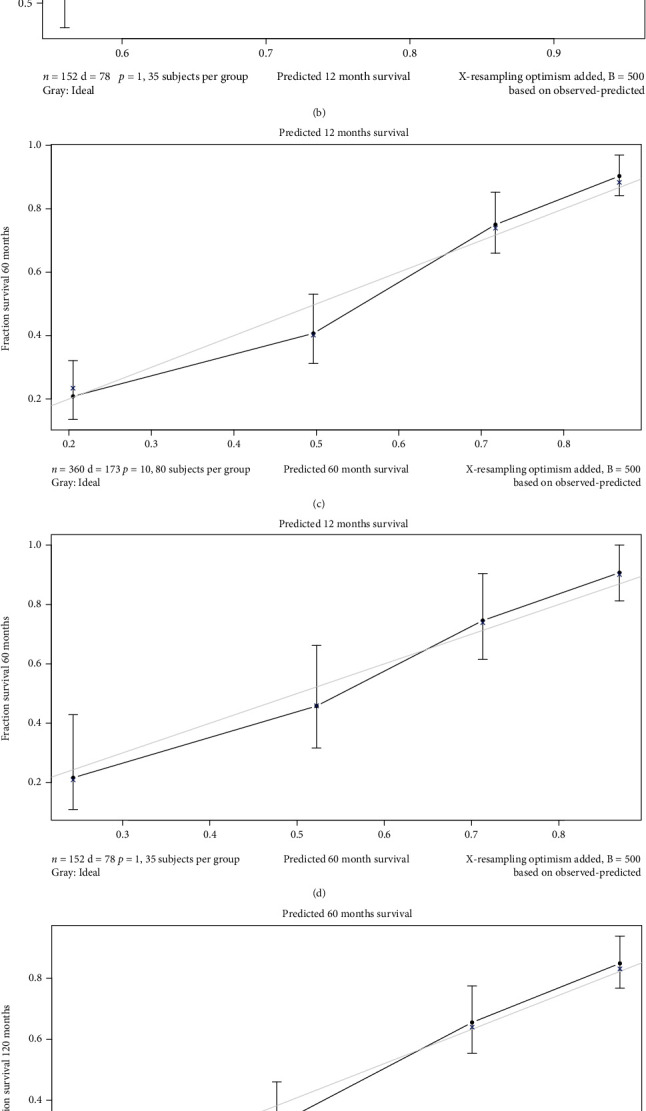
Calibration curves for the nomogram.

**Figure 7:**
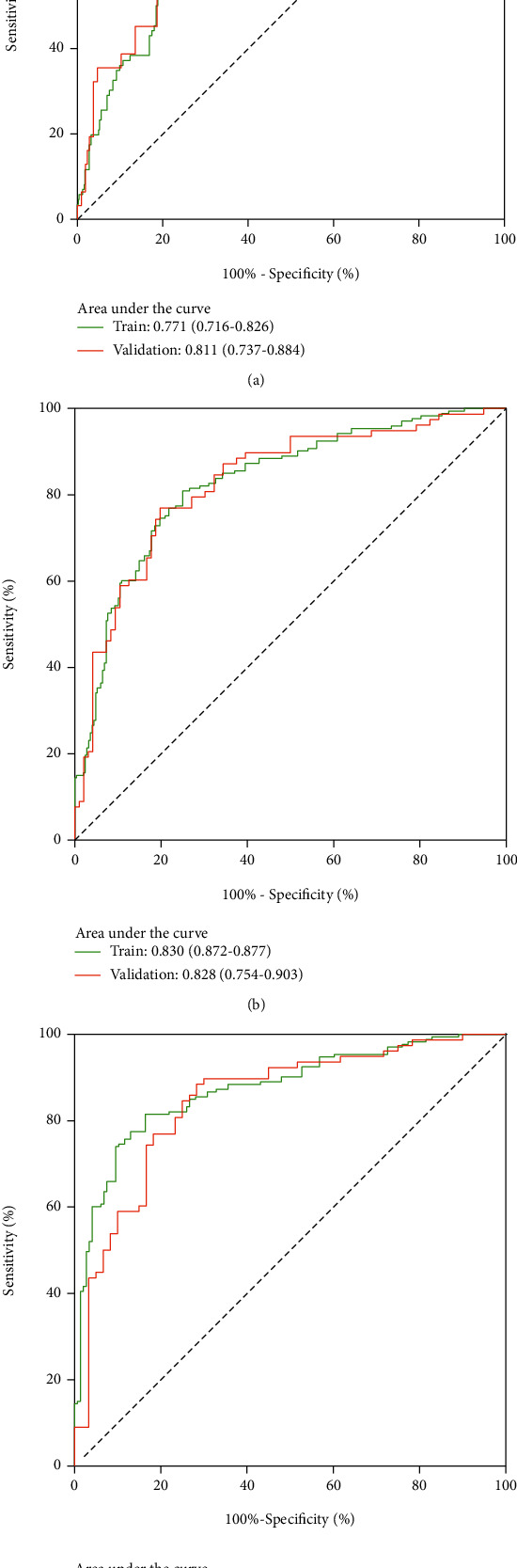
ROC curves for the nomogram.

**Figure 8:**
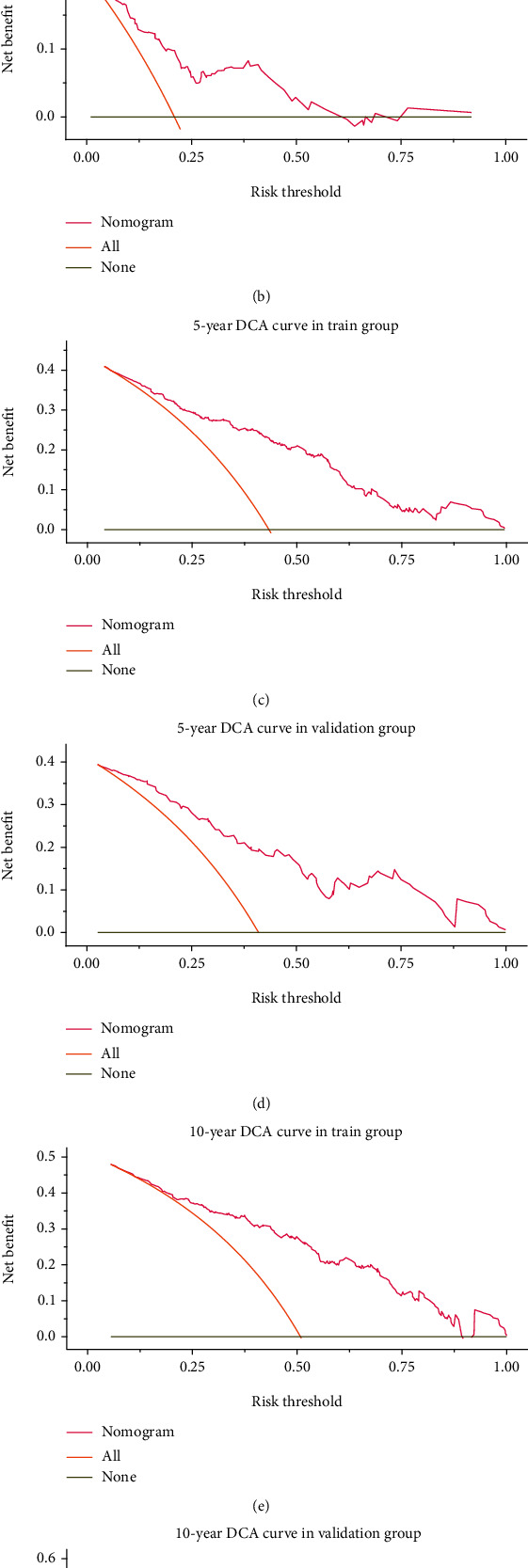
DCA curves for the nomogram.

The bootstrap sampling method was used; that is, the model was internally validated after 500 repeated samplings of the original relevant data in the training group to prevent overfitting. The C-index value was 0.752, suggesting high accuracy. The curve for the prediction accuracy of the nomogram model is shown in the figure. The slope was close to 45°, indicating that the actual and predicted survival rates are in a good agreement; however, the accuracy of the 1-, 5-, and 10-year predictions was different, with a higher accuracy for 5- and 10-year survival probabilities (Figure 6). For time-dependent ROC curves, the AUC values for the 1-, 5-, and 10-year survival rates for the training group were 0.771, 0.830, and 0.874, respectively, and the AUC values for the validation group were 0.811, 0.828, and 0.846, respectively. Because the AUC is close to or greater than 0.8, the model has high differentiation ability and accuracy (Figure 7). To further evaluate the clinical value of the prediction model, clinical DCA curves for 1-, 5-, and 10-year OS were plotted; the net benefit is quite large, suggesting a large beneficial threshold for clinical decision-making based on predictions using this model (Figure 8).

## 4. Discussion

The development of science and technology has led to advancements in medicine. Currently, clinical prediction models are being constructed through the application of more complex methods and larger databases than previously used to establish faster, more accurate, and more universal prediction models. However, the complexity of a model often makes it difficult to interpret and to be applied in daily clinical work. Nomograms play important roles in the current digital age and have been widely used in the prognostic assessment of lung cancer, breast cancer, colorectal cancer, and even rare tumours [7–11]. In our study, we selected EHE patients from the SEER database and screened independent prognostic factors of EHE patients by Cox regression analysis. Through the factors, we established a nomogram prediction model with good prediction ability and wide clinical applicability.

In this study, we analyzed multiple possible prognostic factors in EHE patients, and the results showed that age, tissue differentiation, clinical staging, surgery, chemotherapy, and radiotherapy were all independent factors for patient survival prognosis. In a previous report, females were 4 times as likely to be diagnosed with EHE than were males, and the age range for EHE patients was broad, from children to elderly individuals, with the median age being in the fourth to fifth decade of life [12]. In our study, the age at onset ranged from 2 to 92 years, the median age at onset was 51.5 years, the peak age at onset was 50-59 years, and the prevalence was slightly higher in women than in men (58.9% vs. 41.1%), findings that are generally consistent results in previous reports.

In 2011, the International Hemangioendothelioma, EHE, and Related Vascular Disorders (HEARD) Support Group concluded that the primary sites of EHE were the liver (21%), lung (12%), and bone (14%) [13]. Our study had similar results; the highest proportion (25.6%) of primary tumours occurred in the liver, with a high 5-year OS rate (58.7%). However, the aetiology and pathogenesis of EHE are still not clear but usually considered to be related to factors such as vascular malformations, oral contraceptives, and the abnormal secretion of oestrogen and progesterone [14]. EHE lacks typical clinical manifestations and can metastasize to any region of the body. Approximately 30% of patients experienced distant metastasis, with a 20% mortality rate. In addition, the OS rate for patients with single-lesion tumours was 89%, compared with 50% for patients with more than a single lesion [15].

At present, the conventional treatments are usually radical operation (26%), systemic chemotherapy (24%), debulking surgery (2%), radiation therapy (2%), and others [16]. Some studies have reported that follow-up observation was a reasonable option for patients who were asymptomatic, had stable disease, or were at advanced stages [17, 18]. The treatment approach for most patients with confirmed cases of EHE cases is surgery, and the primary goal is complete resection with negative margins. A retrospective study enrolled 93 EHE patients; approximately 26% of patients with single-focal lesions in the soft tissue, the liver, and other sites who underwent surgery and 75% of patients who received no treatment showed evidence of recurrence at the last follow-up [19]. In addition, in other studies, surgical excision achieved a cure rate of at least 50% [20–22]. In our cohort, patients who underwent surgery had a better prognosis, and the risk of death decreased by 65% compared with that for patients who did not undergo surgery.

A large multicentre retrospective study of the effects of systemic therapy on patients with advanced EHE showed that no relevant antitumour activity for any of the chemotherapeutic drugs was used. A total of 73 patients were included; the median progression-free survival (PFS) was less than 6 months for all patients who received systemic therapy, and the overall response rates (ORRs) were 3% for the anthracycline-based group, 9% for the weekly paclitaxel group, and 4% for the other regimen group [23]. The results from our study also indicated that chemotherapy did not improve patient survival time. Therefore, we look forward to deepening the understanding of the molecular pathogenesis of EHE and helping identify new therapeutic targets.

Due to the inert growth and radiobiological characteristics of EHE, radiotherapy is generally considered to be ineffective for EHE patients, but it is usually used as a means of palliative treatment to alleviate cancer pain caused by bone metastasis. However, in recent years, the European Society for Medical Oncology (ESMO) expert consensus pointed out that radiotherapy can be carefully selected to control symptoms on the basis of the number of lesions, the clinical symptoms of the patient, the tumour burden, and the anatomical location of the primary tumour [24]. In our study, the median OS in the radiotherapy group was 29 months, and the 5-year OS rate was 39.4%, significantly less than that in the no radiotherapy group. We believe that further high-quality studies are essential for selecting an appropriate radiotherapy mode and timing for EHE.

On the basis of Harrell's guidelines, when the outcome is binary, the minimum value of the frequencies of the two response levels should be greater than 10 times the number of predictors. When the outcome is overall survival, the number of deaths is 10 times the number of predictors, so that the expected error in the predicted probabilities from the Cox model is less than 10% [6]. In our study, there were 360 patients in the training group, 152 patients in the validation group, and 251 deaths. In addition, there were 6 predictive variables that meet the standard of Harrell's guidelines.

It is necessary to validate the model's predictive ability, calibration, discrimination, and clinical application. A perfect calibration graph is shown that all observation points (the abscissa axis represents the predicted probability, and the axially of ordinate represents the actual probability) fall on the diagonal, and the distance between the actual observation point and the diagonal represents the absolute error of nomogram prediction results [6]. The calibration graph of our study has good consistency and the value for further verification. The C-index is usually between 0.5 and 1 that defined 0.5-0.7 as low accuracy, 0.71-0.9 as medium accuracy, and greater than 0.9 as high accuracy [25]. The ROC curve is the relationship between sensitivity and specificity. According to the position of the curve, the whole graph is divided to two parts. The area under the curve is used to indicate the accuracy of the prediction [26]. In this study, the C-index is around 0.7, and the AUC is greater than 0.8 which indicate the prediction model has high discrimination ability and accuracy. Although the concept of “net benefit” is not widely adopted now, the threshold interval of benefit is very large through the DCA curve we studied.

Considering the rarity of EHE, large-scale prospective studies are basically impossible to conduct. Therefore, retrospective studies using databases with sufficient sample sizes and relatively detailed patient survival information can be used to provide a basis for strong statistical analyses. To contribute to clinical practice, we established a nomogram that includes all the factors that affect the prognosis of EHE and can better predict the 1-, 5-, and 10-year survival rates for EHE patients, but the data in this study were all from the SEER database; therefore, the selection of cases and variables is a limitation of this study; the clinical data recorded in the SEER database are for U.S. populations, not Asian populations. Second, data on patients' sites of metastasis, regional lymph node invasion, relevant laboratory results, physical condition, and characteristic genetic mutations were lacking; notably, these are very important factors that affect survival and progression. Third, we could not confirm the sequence of surgery, chemotherapy, and radiotherapy, and the dosage regimens for chemotherapy and radiotherapy were unknown. Finally, we were only able to perform internal verification of the data. Due to the rarity of EHE, there are not enough cases from our institution or other institutions for external verification, and further validation using real-world cases and studies is needed. Nevertheless, to date, this is the first EHE study based on the SEER database, and the results are convincing; therefore, this study provides new clinical epidemiological and prognostic information for EHE.

## 5. Conclusion

Our study established the first nomogram prediction model based on the SEER database for EHE patients. The model can predict the 1-year, 5-year, and 10-year survival rates for patients with EHE. Our model incorporates various indicators that can great help for clinical decision-making, so as to realize individual treatment and management of EHE patients.

From our study, we only conduct the internal validation and have not enough cases from our institutions or other institutions for external validation because of the rare cancer, so we need further studies to involve a larger sample size, including more factors to further screen the independent influencing factors of the prognosis of EHE patients and improve the model that provide a reference for the evaluation of the prognosis of EHE patients.

## Figures and Tables

**Table 1:** General clinical characteristics of the EHE patients.

Characteristic	All patients *n* (%)	Training group *n* (%)	Validation group *n* (%)	*χ* ^2^/*t*	*P*
Age				1.608	0.109
Mean ± SD	50.79 ± 17.07	51.57 ± 17.2	48.95 ± 16.67		
Sex				0.000	1.000
Female	300 (58.9)	211 (58.6)	89 (58.6)		
Male	212 (41.1)	149 (41.4%)	63 (41.4%)		
Race				4.836	0.089
Black	56 (10.9)	43 (11.9%)	13 (8.6%)		
Other (Asian)	35 (6.8)	30 (8.3%)	6 (3.9%)		
White	420 (82.0)	287 (79.7%)	133 (87.5%)		
Degree of differentiation				0.962	0.644
Poor (grade III or IV)	47 (9.2)	35 (9.7%)	12 (7.9%)		
Unknown	378 (73.8)	267 (74.2%)	111 (73%)		
Good (grade I or II)	87 (17.0)	58 (16.1%)	29 (19.1%)		
Staging				1.543	0.677
Distant	187 (36.5)	133 (36.9%)	54 (35.5%)		
Localized	146 (28.5)	104 (28.9%)	42 (27.6%)		
Regional	98 (19.1)	64 (17.8%)	34 (22.4%)		
Unknown	81 (15.8)	59 (16.4%)	22 (14.5%)		
Primary location				3.38	0.50
Bone (soft tissue)	121 (23.6)	87 (24.2%)	34 (22.4%)	1	0
Head and neck	40 (7.8)	29 (8.1%)	11 (7.2%)		
Liver	131 (25.6)	84 (23.3%)	47 (30.9%)		
Lung	124 (24.2)	89 (24.7%)	35 (23%)		
Other parts	98 (18.8)	71 (19.7%)	25 (16.4%)		
Surgery				2.117	0.359
No	217 (42.4)	149 (41.4%)	68 (44.7%)		
Unknown	32 (6.3)	26 (7.2%)	6 (3.9%)		
Yes	263 (51.4)	185 (51.4%)	78 (51.3%)		
Chemotherapy				0.076	0.828
No	373 (72.9)	261 (72.5%)	112 (73.7%)		
Yes	139 (27.1)	99 (27.5%)	40 (26.3%)		
Radiotherapy				1.722	0.215
No	392 (76.6)	267 (74.2%)	121 (79.6%)		
Yes	120 (23.4)	93 (25.8%)	31 (20.4%)		

EHE: epithelioid hemangioendothelioma; SD: standard deviation.

**Table 2:** Survival rate and univariate analysis of all EHE patients.

Characteristic	OS					CSS			
	Median (m)	1 y (%)	5 y (%)	10 y (%)	*P*	1 y (%)	5 y (%)	10 y (%)	*P*
All patients	100	76.5	57.4	48.2		77.3	59.0	52.4	
Sex					0.4458				0.2717
Male	97	74.9	55.6	45.1		74.8	55.6	48.3	
Female	120	77.7	58.6	49.6		78.8	61.2	55.1	
Race					0.5487				0.6404
White	100	77.7	59.0	48.5		79.0	60.2	52.4	
Black	100	73.8	51.8	48.1		71.9	53.9	53.9	
Other (Asian)	112	52.4	50.1	43.9		62.0	52.2	52.2	
Staging					<0.0001				<0.0001
Localized	Undefined	88.5	80.5	73.1		89.6	81.7	80.4	
Regional	182	83.2	66.0	57.0		86.5	70.3	64.7	
Distant	29	63.3	37.5	27.4		63.0	38.6	29.6	
Unknown	79	77.7	53.1	42.4					
Degree of differentiation					<0.0001				<0.0001
Good	238	88.4	77.9	72.6		91.6	80.2	74.3	
Poor	19	57.4	37.1	33.7		60.5	40.6	37.0	
Unknown	84	76.1	55.2	44.3					
Primary location					<0.0001				<0.0001
Head and neck	Undefined	84.2	75.6	65.3		91.0	84.8	81.1	
Lung	46	66.5	47.0	37.3		67.9	43.9	37.8	
Liver	199	75.3	58.7	53.8		78.4	61.1	51.7	
Bone (soft tissue)	185	83.7	64.7	53.9		84.1	67.7	66.3	
Other	67	73.3	51.3	38.5					
Surgery					<0.0001				<0.0001
No	34	63.9	40.5	30.4		66.1	42.7	34.0	
Yes	237	88.2	74.0	65.2		88.0	74.8	70.0	
Unknown	17	62.2	34.6	29.6					
Chemotherapy					<0.0001				<0.0001
No	185	82.2	66.5	57.0		83.8	69.4	62.4	
Yes	20	60.9	31.8	20.9		60.5	30.8	22.9	
Radiotherapy					0.0002				0.002
No	135	79.3	62.9	52.1		80.0	63.9	55.8	
Yes	29	67.2	39.4	35.5		67.9	41.9	40.3	

EHE: epithelioid hemangioendothelioma; OS: overall survival; CSS: cancer-specific survival; 1 y: 1 year; 5 y: 5 years; 10 y: 10 years.

**Table 3:** Univariate and multivariate survival analyses of EHE patients in the training group.

Clinical characteristic		Univariate analysis				Multivariate analysis			
		HR	95% CI		*P*	HR	95% CI		*P*
Age		1.029	1.019	1.039	<0.001	1.030	1.019	1.040	<0.001
Degree of differentiation	Poor	Reference			<0.001	Reference			0.046
	Good	0.218	0.112	0.425	<0.001	0.419	0.208	0.845	0.015
	Unknown	0.590	0.379	0.920	0.020	0.781	0.491	1.240	0.294

Staging	Distant	Reference			<0.001	Reference			0.002
	Localized	0.280	0.184	0.425	<0.001	0.505	0.310	0.821	0.006
	Regional	0.368	0.234	0.579	<0.001	0.430	0.263	0.702	0.001
	Unknown	0.526	0.347	0.797	0.002	0.565	0.328	0.974	0.040

Surgery	No	Reference			<0.001	Reference			0.006
	Yes	0.366	0.265	0.506	<0.001	0.616	0.424	0.896	0.011
	Unknown	0.926	0.545	1.573	0.777	1.694	0.853	3.363	0.132

Chemotherapy	Yes vs. no	2.368	1.733	3.235	<0.001	2.123	1.502	3.000	<0.001

Radiotherapy	Yes vs. no	1.913	1.397	2.621	<0.001	1.768	1.266	2.468	0.001

EHE: epithelioid hemangioendothelioma; HR: hazard ratio; CI: confidence interval.

## Data Availability

The data used to support the findings of this study are available from the corresponding authors upon request.
